# The Impact of Diabetes Distress on the Glycemic Control Among Adolescents and Youth With Type 1 Diabetes in Two Tertiary Centers, Jeddah, Saudi Arabia

**DOI:** 10.7759/cureus.17557

**Published:** 2021-08-30

**Authors:** Naseem Y Alyahyawi, Ragad M Alrifay, Norah A Albadi, Maram Y Alqahtani, Raghad M Alzahrani, Bashayr A Nazer, Jumana S Alghamdi, Jumanah A Bahattab

**Affiliations:** 1 Department of Pediatrics, King Abdulaziz University Faculty of Medicine, Jeddah, SAU

**Keywords:** type 1 diabetes (t1d), distress, glycemic control, hba1c, adolescents, saudi, paid, dds

## Abstract

Introduction

Adolescents with type 1 diabetes (T1D) experience multiple symptoms of diabetes distress including fear of acute complications such as severe hypoglycemia which may lead to permanent brain damage or death. They also experience fear of acute hyperglycemia that can lead to diabetic ketoacidosis as well as chronic complication including diabetic nephropathy and retinopathy. No previous research was conducted in Saudi Arabia to assess diabetes distress among adolescents and youth with T1D. This study aimed to assess diabetes distress in adolescents and youth with T1D and its relation to clinical characteristics, glycemic control and diabetes co-morbidities.

Methodology

A cross-sectional study was conducted on 158 patients at King Abdulaziz University Hospital and Dr. Erfan and Bagedo General Hospital, Jeddah, Saudi Arabia. Data about participants’ characters, episodes of DKA, last HbA1c level, diabetes co-morbidities were collected. Diabetes distress (DD) was assessed by the Problem Areas in Diabetes (PAID) and Diabetes Distress Scale (DDS) scores.

Results

The prevalence of diabetes distress among our population of adolescents with T1D was 24.1%. The mean scores of PAID and DDS were 43.56 ± 13.84 and 2.22 ± 1.05, respectively. Patients with suboptimal HbA1c had significantly higher mean PAID and DDS scores. There is also a significant positive correlation between HbA1c level and number of ketoacidosis episodes. A highly significant positive correlation was found between PAID and DDS scores.

Conclusion

This study found that participants with uncontrolled HbA1c had significantly higher mean PAID and DDS scores with a significant positive correlation between the last HbA1c measured level and number of ketoacidosis attacks and PAID and DDS scores. Future studies on larger samples are needed to implement interventions to minimize the burden of diabetes distress among adolescents with T1D.

## Introduction

Diabetes mellitus is a highly prevalent disease with an estimated number of affected individuals reaching 382 million worldwide [1]. The prevalence of diabetes is expected to increase to 583 million by 2035 [1]. Nationally, diabetes is considered the main health problem that affects 24% of the Saudi population [2].

Type 1 diabetes (T1D) is the most common type of diabetes among children and adolescents [3]. There is a significant rise in the incidence of T1D worldwide [3]. According to Diabetes Atlas (8th edition), 35,000 children and adolescents in Saudi Arabia are affected by T1D, which makes Saudi Arabia the 4th country worldwide in terms of the incidence rate (33.5 per 100,000 people) and rank 8th country worldwide in terms of the prevalence of adolescents and children with T1D [4].

T1D is a physically and emotionally demanding disease for both adolescents and youth with T1D [5]. Adolescents with T1D experience different forms of emotional distress related to their illness including fear of acute complications such as severe hypoglycemia, which could lead to permanent brain damage or death and acute hyperglycemia that may lead to diabetes ketoacidosis (DKA) [6]. They also are persistently worried about chronic diabetes complications, i.e., chronic kidney disease, renal failure, diabetic retinopathy and blindness [7].

Adolescence is a transitional period where adolescents go through many psychological and physical changes. This transition period of life leads to multiple psychological stressors according to one study [8]. Adolescents and youth with T1D suffer from additional distress because of having to deal with the daily ongoing demands of managing T1D while going through this critical period of life during the transition to adult life [9]. This fact leads to the development of the term: “Diabetes Distress”, which is defined as the negative emotional impact of living with diabetes [10].

According to one study, adolescents with T1D who reported diabetes distress indicate that worrying about the future and the possibility of suffering from serious complications was the most common source of their diabetes distress [7]. They also feel overwhelmed by their diabetes regimen and feel angry when thinking about living with diabetes [7]. Identifying diabetes distress has a very important clinical value since many studies reported a strong correlation between reporting diabetes distress and deteriorating self-care and poor glycemic control in adolescents and youth with T1D [11].

A study was conducted at the University of Florida found a positive correlation between HbA1c and diabetes distress, such that those adolescents who reported poor glycemic control had a higher score in the Diabetes Distress Scale (DDS) indicating a higher level of emotional distress related to diabetes [12].

There is a wealth of evidence that addressed the impact of diabetes distress on adults with diabetes. For example, one study reported a higher prevalence of diabetes distress among adults with T1D who reported lower quality of life and had poor glycemic control [13]. Other studies were conducted locally to assess diabetes distress among adults with type 2 diabetes mellitus (T2DM) in Saudi Arabia reported higher diabetes distress among individuals with poor glycemic control in one study [14]. The challenge of controlling diabetes regimen was reported as the main source of diabetes distress among adults with T2DM who participated in another study [15].

There was no previous study conducted to assess diabetes distress among adolescents and youth with T1D in Saudi Arabia. Thus, this study aimed to assess the relationship between the diabetes distress and the glycemic control among adolescents and youth with T1D and its relation to patient’s characters, glycemic control (HbA1c) level and the presence of diabetes co-morbidities.

## Materials and methods

This cross-sectional study was done from January 2019 to December 2020 and was approved by Biomedical Ethics Research Committee in King Abdulaziz University (approval number: 704-19; approval date: December 02, 2019). We recruited 158 participants from the pediatric and adult diabetes and endocrinology clinics at King Abdulaziz University Hospital and Dr. Erfan and Bagedo General Hospital, Jeddah, Saudi Arabia.

The inclusion criteria included adolescents and youth with T1D, with an age between 10 and 19 years and youth age between 19 and 24 years, who spoke Arabic. All participants had at least one HbA1c measured during their follow-up (controlled defined as HbA1c <7.5% and uncontrolled as HbA1c >7.5%) and they provided written consent. We excluded all patients with T1D who were diagnosed with T1D <6 months prior to recruitment, T1D patients with developmental delay, autism or diagnosed mental health conditions such as depression or eating disorders and patient who could not provide a written informed consent.

The study team approached any adolescent or youth with T1D who met the eligibility criteria who attended the clinic. The study team explained the study aim and explained the elements of the consent sheet. Adolescents who agreed to participate signed the consent sheet prior to their participation. Younger adolescents (10-11 years) provided assent to participate in the study.

A predesigned checklist was prepared to collect data about participants’ demographic information: age, sex, nationality, income, parental level of education, parental marital status, number of previous episodes with DKA, last 3 HbA1c levels. In addition to data about diabetes co-morbidities (hypothyroidism, hypertension, celiac disease, kidney and eye disorders).

The second section of the checklist included the Problem Areas in Diabetes (PAID) [16], Diabetes Distress Scale (DDS) [17]. The DDS is a 17- item questionnaire that uses a Likert scale with each item, scored from 1 (no distress) to 6 (serious distress). A mean item score of ≥ 3 was taken as a level of distress worthy of clinical attention. The PAID scale is another tool for assessing diabetes distress in patients with diabetes. It consists of a 20-item questionnaire that measures diabetes-related emotional distress, and involves a range of negative emotional problems of diabetic patients [18]. Statements are given a score ranging from 0 (not a problem) to 4 (a serious problem), where higher scores indicate higher levels of diabetes-related distress.

A native Arabic speaker with high English proficiency and a medical background translated the questionnaires from English to Arabic. Bilingual experts evaluated the translations for logic and clarity. The questionnaires were then given to a native English speaker with high Arabic proficiency to translate back to English. The final English versions were compared to the original English questionnaires for accuracy. Arabic questionnaires were pre-tested using a focus group similar to the final sample group to ensure all questions were clear and unambiguous [15].

Data were analyzed using (SPSS) version 25 (IBM Corp., Armonk, NY). The mean and standard deviation (mean ± SD) are reported for continuous variables. While categorical variable was expressed as numbers and percentages, and quantitative data were expressed as mean and standard deviation. For qualitative data, independent sample t-test and one-way ANOVA tests were applied for parametric variables and Mann-Whitney and Kruskal-Wallis tests were applied for non-parametric variables. Correlation analysis was done using the Spearman’s test for non-parametric variables and Pearson’s test for parametric variables. A p-value of <0.05 was considered statistically significant.

## Results

The mean age of the study’s participants was 15.36 ± 3.99 years (Table 1). Of those, 57.6% were females and 66.5% had a Saudi nationality. 33.5% had a secondary school education. The mean age at the onset of T1D was 8.84 ± 4.26, mean T1D duration was 6.51 ± 5.05, mean last HbA1c level and mean number of ketoacidosis episodes were, 9.88 ± 2.77 and 1.75 ± 2.79, respectively. 60.1% of the study group had a rented house and 43% had a monthly income < 5000 SR. Of the participants, 4.4%, 15.8%, 3.2%, 25.3% and 7% had a celiac disease, hypothyroidism, hypertension, eye disorders and kidney disorders, respectively. 12.7% of the participants had separated parents.

**Table 1:** Distribution of the participants according to their characters, chronic diseases, diabetes duration, age at diagnosis, HbA1c and number of ketoacidosis episodes (Number: 158).

Variable	No. (%)
Age	15.36± 3.99
Gender	
Female	91 (57.6)
Male	67 (42.4)
Nationality	
Saudi	105 (66.5)
Non-Saudi	53 (33.5)
Educational level	
Primary	40 (25.3)
Intermediate	36 (22.8)
Secondary	53 (33.5)
University	29 (18.4)
Habitat	
Rent	95 (60.1)
Owner	63 (39.9)
Monthly income	
<5,000	68 (43)
5,000-10,000	50 (31.6)
11,000-20,000	32 (20.3)
>20,000	8 (5.1)
Separated parents	
No	138 (87.3)
Yes	20 (12.7)
HbA1C	
Controlled	32 (20.3)
Not controlled	126 (79.7)
Age at diabetes mellitus diagnosis	8.84 ± 4.26
Diabetes mellitus duration	6.51 ± 5.05
Last HbA1c measured level	9.88 ± 2.77
Number of ketoacidosis attacks	1.75 ± 2.79
Emergency unit visits last year	
No	86 (54.4)
Yes	72 (45.6)
Celiac disease	
No	151 (95.6)
Yes	7 (4.4)
Hypothyroidism	
No	133 (84.2)
Yes	25 (15.8)
Hypertension	
No	153 (96.8)
Yes	5 (3.2)
Eye disorders (including diabetes retinopathy)	
No	118 (74.7)
Yes	40 (25.3)
Diabetic nephropathy	
No	147 (93)
Yes	11 (7)
Other diseases	
No	137 (66.7)
Yes	21 (13.3)

**Figure 1:**
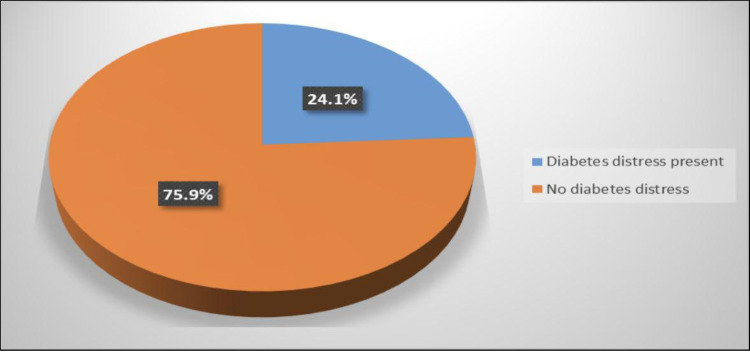
Percentage distribution of the prevalence of diabetes distress among participants.

The mean scores of PAID and DDS were 43.56 ± 13.84 and 2.22 ± 1.05, respectively. Figure 1 shows that the prevalence of participants who had a DDS ≥ 3 indicating a level of distress worthy of clinical attention was 24.1%. Table 2 shows that a significant positive correlation was found between poor glycemic control (HbA1c level >7.5%) DDS and PAID score (p = 0.004) The table also showed a significant positive correlation between number of ketoacidosis episodes, and PAID and DDS scores (p = 0.002). There were no correlations between the presence of diabetes co-morbidities (hypothyroidism, wheat allergy and hypertension) and diabetes complications including diabetic retinopathy and nephropathy and DDS and PAID score (Table 3 and Table 4). There was also no correlation between DDS and PAID score and patients’ age, nationality, income and parental education or marital status. 

**Table 2:** Spearman correlation analysis between Problem Area in Diabetes, Diabetes Distress Scale and participants age, age at diagnosis, diabetes mellitus duration, last HbA1c measured level and number of ketoacidosis episodes.

Problem Areas in Diabetes (PAID)		
	r	p-value
Age	0.05	0.504
Age at diagnosis	0.05	0.491
Diabetes mellitus duration	0.08	0.302
Last HbA1c level	0.22	0.004
Ketoacidosis number	0.24	0.002
Diabetes Distress Scale (DDS)		
	r	p-value
Age	0.13	0.1
Age at diagnosis	0.01	0.898
Diabetes mellitus duration	0.11	0.14
Last HbA1c measured level	0.24	0.002
Ketoacidosis number	0.23	0.002

**Table 3:** Relationship between participants’ characters, chronic diseases and having separated parents and mean Problem Area in Diabetes scores. *Independent sample t-test. **One-way ANOVA test. ***Mann-Whitney test.

Variable	Problem Areas in Diabetes (PAID) (mean ± SD)	Test	p-value
Gender			
Female	43.68 ± 14.12	0.09*	0.73
Male	43.48 ± 13.71		
Nationality			
Saudi	43.87 ± 14.13	0.39*	0.551
Non-Saudi	42.96 ± 13.35		
Educational level			
Primary	45.75 ± 15.06	2.09**	0.1
Intermediate	40.02 ± 12.12		
Secondary	45.92± 13.69		
University	40.65 ± 13.56		
Habitat			
Rent	45.02 ± 14.04	1.62*	0.905
Owner	41.38 ± 13.34		
Monthly income			
< 5,000	43.42 ± 14.33	0.59**	0.621
5,000-10,000	44.9 ± 14.35		
11,000-20,000	43.18 ± 12.47		
>20,000	38 ± 12.2		
Separated parents			
No	46.45 ± 14.1	0.99*	0.784
Yes	43.15 ± 13.8		
Celiac disease			
No	38.71 ± 14.3	0.94*	0.894
Yes	43.79 ± 13.82		
Hypothyroidism			
No	39.56 ± 12.32	1.58*	0.211
Yes	44.32 ± 14.02		
Eye disorders			
No	43.97 ± 13.8	0.21*	0.937
Yes	43.43 ± 13.9		
Diabetic nephropathy			
No	44.45 ± 16.1	0.21*	0.707
Yes	43.50 ± 13.71		
Other diseases			
No	43.04 ± 14.86	0.18*	0.979
Yes	43.64 ± 13.73		

**Table 4:** Relationship between participants’ characters and chronic diseases and mean Diabetes Distress Scale scores. * Mann-Whitney test. ** Kruskal-Wallis test.

Variable	Diabetes Distress Scale (DDS) (mean ± SD)	Test	p-value
Gender			
Female	2.20 ± 1.09	0.42*	0.671
Male	2.23 ± 1.03		
Nationality			
Saudi	2.27 ± 1.09	0.78*	0.429
Non-Saudi	2.11 ± 0.98		
Educational level			
Primary	2 ± 0.99	3**	0.162
Intermediate	2.09 ± 1.01		
Secondary	2.43 ± 1.11		
University	2.29 ± 1.07		
Habitat			
Rent	2.23 ± 1.03	0.27*	0.785
Owner	2.20 ± 1.1		
Monthly income			
<5,000	2.17 ± 1.02	3**	0.793
5,000-10,000	2.31 ± 1.02		
11,000-20,000	2.18 ± 1.17		
>20,000	2.17 ± 1.24		
Separated parents			
No	2.31 ± 1.08	0.48*	0.63
Yes	2.20 ± 1.05		
Celiac disease			
No	1.63 ± 0.51	1.34*	0.18
Yes	2.25 ± 1.07		
Hypothyroidism			
No	2.24 ± 1.04	0.26*	0.793
Yes	2.21 ± 1.06		
Eye disorders			
No	2.29 ± 0.99	0.84*	0.398
Yes	2.19 ± 1.08		
Diabetic nephropathy			
No	2.70 ± 1.28	1.37*	0.17
Yes	2.18 ± 1.03		
Other diseases			
No	2.55 ± 1.26	1.38*	0.167
Yes	2.17 ± 1.01		

**Figure 2:**
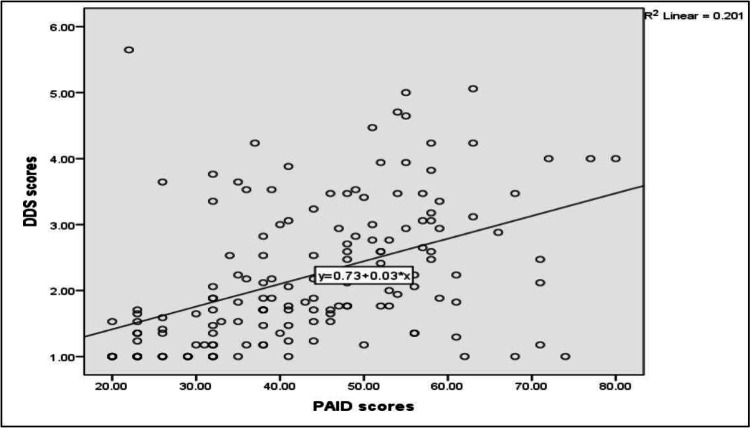
Spearman correlation analysis between Problem Areas in Diabetes scores and Diabetes Distress Scale scores. (r= 0.52, p-value= < 0.001)

 Figure 2 shows that a highly significant positive correlation was found between PAID scores and DDS scores (p=< 0.05). 

## Discussion

This study aimed to assess diabetes distress in adolescents and youth with T1D and its relation to patient’s characters, glycemic control (HbA1c level), and presence of diabetes co-morbidities in Saudi Arabia.

It was found that patients with uncontrolled HbA1c had a significant higher PAID and DDS scores. This study is consistent with a previous systematic review which showed high prevalence of diabetes distress among adolescents with T1D and identified a positive correlation between suboptimal glycemic control and diabetes distress among adolescents with T1D [19].

 This study is also consistent with findings in previous study which utilized PAID score to estimate the relationship between diabetes distress and glycemic control in 3489 individuals with T1D or T2D. PAID score quartile was markedly correlated with poor glycemic control (HbA1c ≥53 mmol/mol [7.0%]), with a significant linear trend (p = 0.03) [20].

A high prevalence of adolescents with T1D with low economic status was identified in our population of patients with T1D reaching 43%.

The relationship between diabetes distress and diabetes duration was not consistent in the literature. In one previous study, a significant positive correlation between PAID scores and diabetes duration was reported in which that longer diabetes duration was associated with higher PAID score [21]. We haven’t identified a significant correlation between PAID scores and T1D duration, our finding endorsed other findings from previous literature [22,23]. This study found a significant positive correlation between PAID scores and DDS scores.

A limitation of this study is the self-administered questionnaire used that may have a recall bias.

## Conclusions

This study found that participants with uncontrolled HbA1c had significant higher mean PAID and DDS scores with a significant positive correlation between last HbA1c measured level and number of ketoacidosis episodes and PAID and DDS scores. A highly significant positive correlation was found between PAID and DDS scores. There is a need for future studies assessing diabetes distress among Saudi adolescents and youth with Type 1 Diabetes that include a larger sample. Results of these studies will help in improving the understanding of the trend and the causes of diabetes distress among adolescents with T1D in Saudi Arabia, this is important step to suggest and implement interventions to minimize the effect of diabetes distress among this age group.

## Appendices

                       THE DIABETES DISTRESS SCREENING SCALE

DIRECTIONS: Living with diabetes can sometimes be tough. There may be many problems and hassles concerning diabetes and they can vary greatly in severity. Problems may range from minor hassles to major life difficulties. Listed below are two potential problem areas that people with diabetes may experience. Consider the degree to which each of the two items may have distressed or bothered you DURING THE PAST MONTH and circle the appropriate number.

Please note that we are asking you to indicate the degree to which each item may be bothering you in your life, NOT whether the item is merely true for you. If you feel that a particular item is not a bother or a problem for you, you would circle''1''. If it is very bothersome to you, you might circle "6".

Not a Problem = 1 A Slight Problem = 2 A Moderate Problem = 3 Somewhat Serious Problem = 4 A Serious Problem = 5 A Very Serious Problem = 6

1. Feeling that diabetes is taking up too much of my mental and physical energy every day. 2. Feeling that my doctor doesn't know enough about diabetes and diabetes care. 3. Feeling angry, scared, and/or depressed when I think about living with diabetes. 4. Feeling that my doctor doesn't give me clear enough directions on how to manage my diabetes. 5. Feeling that I am not testing my blood sugars frequently enough. 6. Feeling that I am often failing with my diabetes routine. 7. Feeling that friends or family are not supportive enough of self-care efforts (e.g. planning activities that conflict with my schedule, encouraging me to eat the "wrong" foods). 8. Feeling that diabetes controls my life. 9. Feeling that my doctor doesn't take my concerns seriously enough. 10. Not feeling confident in my day-to-day ability to manage diabetes. 11. Feeling that I will end up with serious long-term complications, no matter what I do. 12. Feeling that I am not sticking closely enough to a good meal plan. 13. Feeling that friends or family don't appreciate how difficult living with diabetes can be. 14. Feeling overwhelmed by the demands of living with diabetes. 15. Feeling that I don't have a doctor who I can see regularly enough about my diabetes. 16. Not feeling motivated to keep up my diabetes self management. 17. Feeling that friends or family don't give me the emotional support that I would like.

                    Problem Areas in Diabetes Questionnaire (PAID)

The Problem areas in diabetes (PAID) questionnaire is a psychometrically sound tool for detecting diabetes-related distress. The PAID questionnaire includes 20 items, each of which focuses on a different commonly experienced problem with diabetes. Patients indicate how much each issue affects them personally, on a scale of 0 (not a problem) to 4 (serious problem). Individual items scored ≥3 (indicating a somewhat serious or serious problem area) should be discussed with the patient.

Not a problem = 0 Minor problem = 1 Moderate problem = 2 Somewhat serious problem = 3 Serious problem = 4

1. Not having clear and concrete goals for your diabetes care? 2. Feeling discouraged with your diabetes treatment plan? 3. Feeling scared when you think about living with diabetes? 4. Uncomfortable social situations related to your diabetes care (e.g., people telling you what to eat)? 5. Feelings of deprivation regarding food and meals? 6. Feeling depressed when you think about living with diabetes? 7. Not knowing if your mood or feelings are related to your diabetes? 8. Feeling overwhelmed by your diabetes? 9. Worrying about low blood sugar reactions? 10. Feeling angry when you think about living with diabetes? 11. Feeling constantly concerned about food and eating? 12. Worrying about the future and the possibility of serious complications? 13. Feelings of guilt or anxiety when you get off track with your diabetes management? 14. Not “accepting” your diabetes? 15. Feeling unsatisfied with your diabetes physician? 16. Feeling that diabetes is taking up too much of your mental and physical energy every day? 17. Feeling alone with your diabetes? 18. Feeling that your friends and family are not supportive of your diabetes management efforts? 19. Coping with complications of diabetes? 20. Feeling “burned out” by the constant effort needed to manage diabetes?
